# The feasibility of targeted axillary dissection for breast cancer axillary surgery de-escalation after neoadjuvant therapy: a prospective cohort study

**DOI:** 10.1097/JS9.0000000000002058

**Published:** 2024-08-22

**Authors:** Qing-Da Fan, Zhao Bi, Li-Guo Gong, Peng Chen, Bin-Bin Cong, Bao-wei Duan, Yu-Guang Chen, Peng-Fei Qiu, Yong-Sheng Wang

**Affiliations:** a Department of Breast Cancer Center, Shandong Cancer Hospital and Institute, Shandong First Medical University and Shandong Academy of Medical Sciences, Jinan; b Department of Breast Surgery, Yantaishan Hospital of Yantai city, Yantai; c Department of Breast Surgery, Taian Tumor Hospital, Taian, People’s Republic of China

**Keywords:** breast cancer, neoadjuvant therapy, sentinel lymph node biopsy, targeted axillary lymph node dissection, tracers

## Abstract

**Purpose:**

Targeted axillary dissection (TAD) after neoadjuvant therapy (NAT) includes the removal of both marked and sentinel lymph nodes (SLNs). The aim was to investigate the optimization of TAD localization techniques after NAT in breast cancer patients.

**Methods:**

From November 2020 to 2022, the authors prospectively enrolled 107 lymph node-positive breast cancer patients at Shandong Cancer Hospital, all of them received complete cycles of NAT. Before treatment, patients were randomly divided into three groups: group A, marked node with clip (*n*=34); group B, marked node with ^125^I seed (n=32); and group C, marked node with both a clip and ^125^I seed (*n*=41). Dual tracers were used to search for SLNs after NAT. The primary endpoint was the detection rate of marked nodes and false-negative rate (FNR).

**Results:**

The detection rates using the TAD localization technique were 82.6% (28/34), 100% (32/32), and 100% (41/41) for groups A, B, and C, respectively (*P*>0.05). The FNR rates were 15.8%, 5.9%, and 5.6% among groups A, B, and C, respectively (*P*>0.05). The FNR rates in cN1 patients were 5.1%, 2.7%, and 2.6%, among these three groups, respectively (*P*>0.05). The change in distance between ^125^I seeds and clips in axillary lymph nodes was <3 mm. The FNR rates of TAD guided by dye tracers, radiolabeled tracers, and dual tracers were 5.4%, 5.2%, and 3.4%, respectively (*P*>0.05). The negative predictive values were 93.0%, 93.0%, and 95.2%, respectively (*P*>0.05).

**Conclusion:**

Considering the inexpensive and high detection rate of ^125^I seeds, it is recommended to use ^125^I seeds for localizing metastatic nodes in neoadjuvant setting. The TAD guided by dye tracer is also a feasible option for axillary de-escalation surgery after NAT in countries or regions without access to radiolabeled colloid.

## Introduction

Highlights

- The aim was to investigate the optimization of TAD localization techniques.
- Patients with cN1 breast cancer are an appropriate population for sentinel lymph node biopsy after NAT.
- The TAD guided by dye tracer is also feasible for axillary de-escalation surgery after NAT in country or region without radiolabeled colloid.

Neoadjuvant therapy (NAT) in breast cancer patients helps downstage tumors to achieve breast conservation and eliminate axillary lymph node dissection (ALND)^1^. NAT could facilitate de-escalation management of the axillary region in breast cancer patients, and the survival benefit has been confirmed of initial cN1 patients with sentinel lymph node (SLN)-negative omitting ALND after NAT^2^. The main thrust of current research focuses on how to reduce the false-negative rate (FNR) of sentinel lymph node biopsy (SLNB) after NAT. In contrast, there is a lack of large samples studies on the effectiveness of SLNB after NAT in patients with cN2 disease, which cannot accurately predict residual axillary tumor burden and clarify the risk of postoperative axillary recurrence and metastasis.

Targeted axillary dissection (TAD) is a precise surgical technique that involves placing markers on positive axillary lymph node (ALN) and detect this node after NAT, which could further reduce the FNR of SLNB^3–5^ and improves pathologic evaluation for residual nodal disease after NAT. TAD is thought to be a safe and reliable axillary staging technique, thereby eliminating the need for ALND and postoperative radiotherapy. The 2024 V2 NCCN Guideline recommend the detection of ≥3 SLNs with dual tracers, or marking and detecting positive lymph nodes intraoperatively to reduce the FNR of SLNB after NAT (Class IIB recommendation)^6^. However, the populations who may benefit from TAD have not been identified according to evidence-based medicine. The best marker localization technique for positive lymph node has not been optimized. Considering the lack of radiolabeled colloid in some countries or regions, how to jointly apply tracers and TAD techniques to improve the accuracy of SLNB is also the direction of current research after NAT.

Therefore, the aim of this study was to determine the best TAD localization marker, evaluate the feasibility and accuracy of SLNB guided by different TAD techniques after NAT, and identify the populations who will likely benefit from axillary de-escalation surgery after NAT.

## Patients and methods

### Patients

From November 2020 to 2022, we prospectively enrolled 107 lymph node-positive breast cancer patients who received NAT in Shandong Cancer Hospital. Eligible patients had cT1-3N1-2M0 disease according to the WHO criteria, and completed a full cycle of NAT. Patients were not eligible if they had a medical history of previous malignancy, bilateral breast cancer, had T4 or N3 disease, or had received treatment of the axilla by surgery or radiotherapy. The study was approved by the Shandong Cancer Hospital Ethics Committee (No. SDTHEC20200324). Informed consents were obtained from all patients, and all procedures were in accordance with the ethical standards of the responsible institutional committee on human experimentation and with the Helsinki Declaration. The work has been reported in line with the strengthening the reporting of cohort, cross-sectional, and case–control studies in surgery (STROCSS) criteria^7^.

### Tracer

In our center, we used the dual-tracers to search SLNs combined with dye tracer and radiolabeled tracer. Radiolabeled tracer were injected using ‘modified injection technique’, in which 18-37 MBq of ^99m^Tc-labeled colloid (1–2 ml) was injected into the glands around the areola at the 6-point and 12-point sites under ultrasound guidance for 3–18 h prior to surgery^8,9^. Dye tracer was injected subcutaneously into the primary tumor 20 min before surgery (4 ml in mastectomy patients and 2 ml in breast-conserving patients).

### Research subgroups

One of two radiologists who had 10 and 6 years of experience in breast ultrasound (US) respectively performed preoperative axillary US with Siemens S2000 ultrasound scanner (Siemens Healthineers) equipped with a 4–9 MHz linear array transducer. After performing whole-breast US, the same radiologists performed axillary US routinely and recorded suspicious US features of the axillary lymph node (ALN). If patients had multiple suspicious ALNs on the US, the largest ALN was selected for accessing by fine-needle aspiration (FNA) biopsy prior to treatment according to the wishes of doctors and patients. Repeated negative pressure aspiration was performed for the abnormal parts of the ALN guided by ultrasound, and the biopsy specimens were examined by cytology.

**Figure 1:**
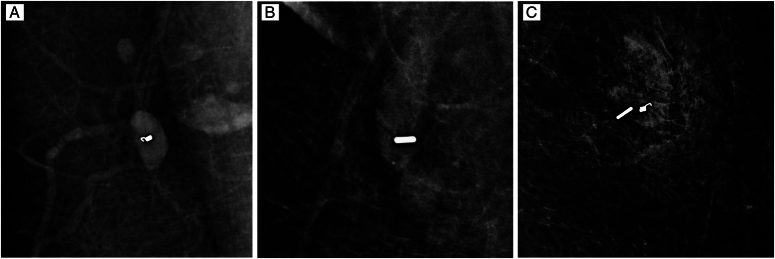
TAD technology optimization process and ^125^I seed implantation process. A: Metal marker clips were placed in metastatic axillary lymph nodes before NAT under ultrasound guidance; B: ^125^I seed alone. C: Both metal marker clips and ^125^I seed.

**Figure 2:**
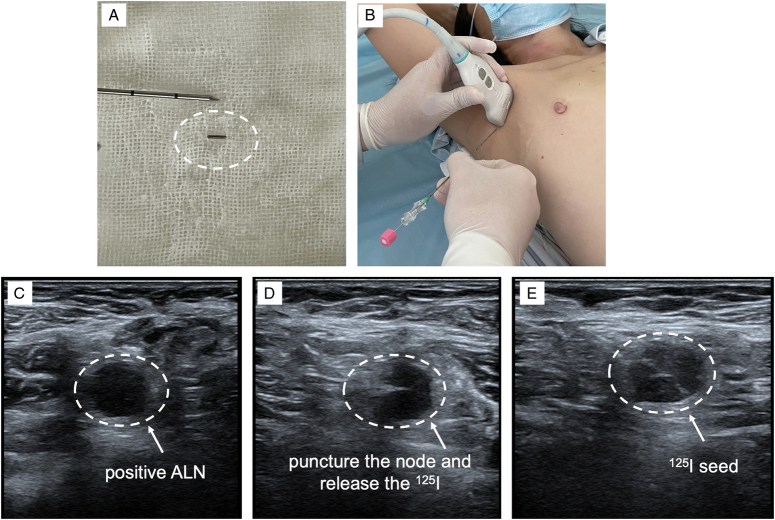
A, Puncture Needle 17 g×10 cm; B, Surgical operations of TAD technology; C, The suspicious lymph node on the US; D, Puncture the node and release ^125^I seed; E, Marked lymph node.

**Figure 3:**
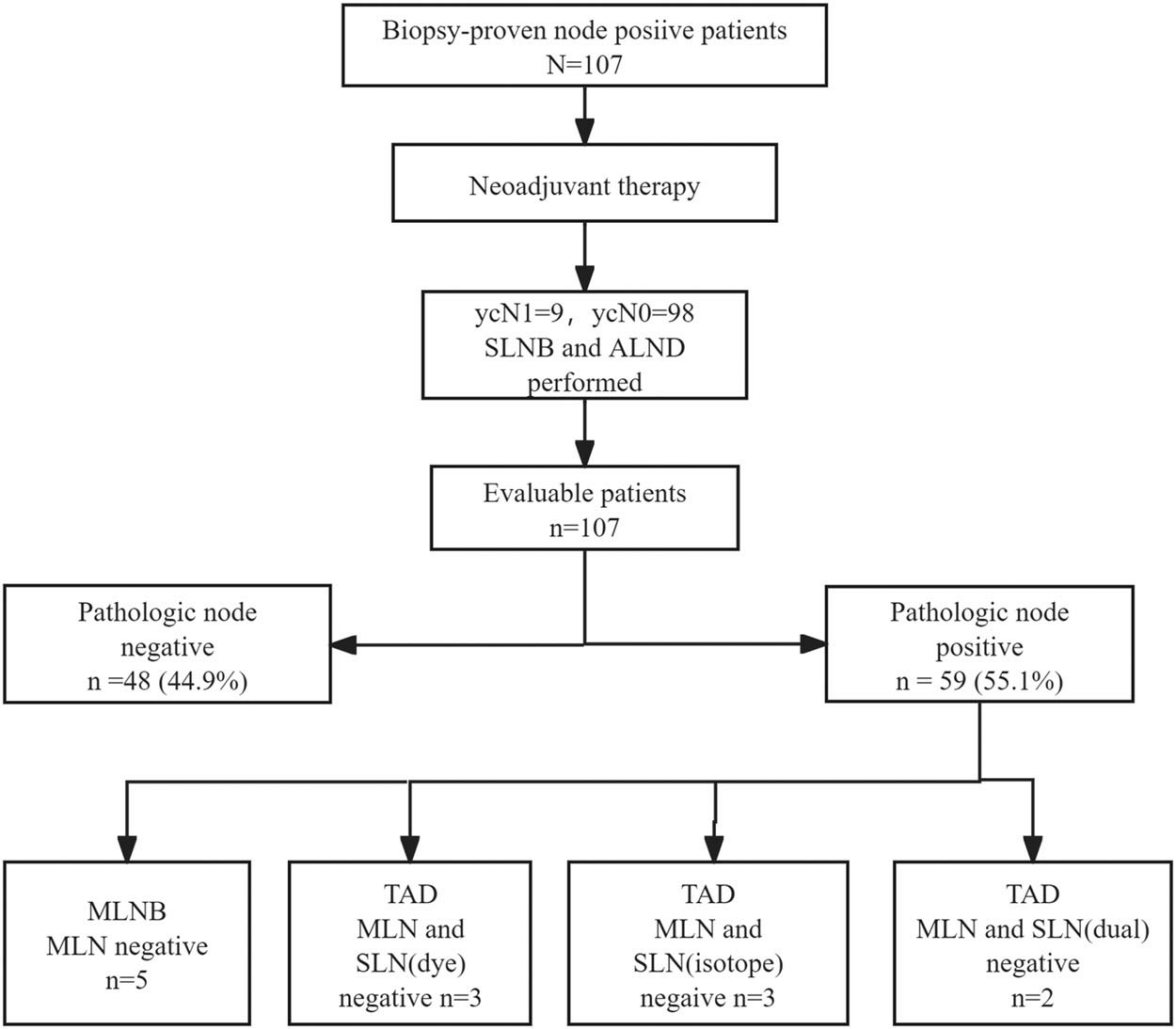
Study flowchart of our study.

For patients with FNA positive, patients were randomly divided into three groups (Fig. 1). Group A: metal marker clip (ultrasound-enhanced coils) was placed into a positive marked lymph node (MLN) under ultrasound guidance before NAT. The presence of metal marker clips was confirmed by intraoperative dissection of the SLN for observation or X-ray radiography. Group B: ^125^I seeds consisting of a silver wire source core labeled with iodine nuclide (^125^I) and a medical titanium alloy tube at a dose of 0.1–0.3 mCi were placed in the positive ALN before NAT (Figs. 2 and 3). The MLN was targeted for detection. Group C: ^125^I seed and metal marker clip were simultaneously placed in the positive ALNs. The MLNs were removed intraoperatively using a gamma probe.

### Axillary surgery

All patients received SLNB followed by ALND. To verify the accuracy of the different tracers guided by the TAD technique, SLNs detected by different tracers were recorded and counted. SLNs detected by dye tracer are designated as dye SLNs and SLNs detected by radiolabeled tracer are designated as radiolabeled SLNs. Three TAD procedures were recorded based on the blue-stained lymph vessel and radionuclide counts for each detected lymph node, as follows: dye tracer TAD; radionuclide tracer TAD; and dual tracer TAD. The feasibility and accuracy of the three different tracer TAD techniques were determined in predicting the status of ALN after NAT. The detected lymph nodes were subjected to intraoperative print cytology and frozen section examination.

### Pathological evaluation

Each SLN was examined at multiple histologic levels. Tumor deposits were categorized as isolated tumor cells (ITC) (<0.2 mm), micro-metastases (0.2–2 mm), or macro-metastases (>2 mm). In this study, we defined macro-metastases, micro-metastases, and ITC as positive lymph nodes.

### Data analysis

All statistical data were analyzed with SPSS version 26.0 software. and the *χ* ^2^ test or Fisher exact test or paired *t*-test was used for intergroup comparisons of count data. A *P*<0.05 was considered statistically significant.

## Result

### The characteristics of enrolled patients

The clinical-pathological features were summarized in Table 1. There were 107 patients underwent successful TAD (Fig. 3). There were 34, 32, and 41 patients placed marker clip, ^125^I seed, and marker clip combined with ^125^I seed, respectively. And there were 83 and 24 patients had cN1 and cN2 disease, respectively. All patients were performed SLNB using dual-tracers.

**Table 1:** Clinicopathological characteristics of enrolled patients.

Characteristic	*n* (%)
Median age, years	47, range 24–69
Clinical T stage	
T1	15 (14.0)
T2	68(63.6)
T3	24 (22.4)
Clinical LN stage before NAT	
cN1	83(77.6)
cN2	24(22.4)
Clip location - no.	
Sentinel node	73(80.2)
Non-sentinel node	18(19.8)
bpCR	
Yes	51(47.7)
No	56(52.3)
ypN0	
Yes	57(53.3)
No	50(46.5)
Molecular subtype	
HR+/HER2-	32(29.9)
TNBC	12(11.2)
HER2+(HR-)	33(30.80)
HER2+(HR+)	30(28.1)
Ki67	
＜20%	27(25.2)
≥20%	80(74.8)
ALND	107(100)

### The accuracy of TAD after NAT

#### The FNR for SLNB guided by TAD technology

The overall FNR rate for MLN was 9.3% (5/54). The FNR rates for applying marker clip, ^125^I seeds, and combined marker for localizing metastatic lymph nodes were 5.9% (1/17), 5.6% (1/18), and 15.8% (3/19), respectively (*P*=0.06). The negative predictive values were 94.1% (16/17), 92.9% (13/14), and 92.7% (38/41), respectively (*P*=0.981). The inconsistency rates of MLN and SLN under the three TADs were 29.4% (10/34), 21.9% (7/32), and 9.5% (8/41), respectively (*P*=0.59). The detection rate was 82.6, 100, and 100% for the application of a marker clip, ^125^I seeds, and combined marker clips to localize positive ALN.

#### Displacement of ^125^I seed within lymph nodes and breast tumors

Intraoperative lymph node dissection showed that the ^125^I seeds were in the same lymph node as the marker clips (Table 2). If the change of distance between the ^125^I seeds and the marker clips before and after NAT was <3 mm, then we defined the ^125^I seeds as not displaced in lymph nodes and breast tumors. We calculated the difference in relative distance of ^125^I seed and clip before and after NAT by oblique mammography, and found that the change of distance between ^125^I seeds and clip before and after NAT was <3 mm in 100% (8/8) and 100% (27/27) in ALNs and breast tumors, respectively. Axial mammography showed that there were 96.0% (24/25) of breast tumors had a change <3 mm distance between ^125^I seeds and clips before and after NAT. Therefore, we believed that there was no displacement between ^125^I seeds and clips placed in ALN.

**Table 2:** Displacement of ^125^I seed in lymph nodes and breast tumors

		S^※^	
	ΔS^※^<3 mm %(n)	Before NAT (mm)	After NAT (mm)
ALN			
CC	100% (8/8)^a^	9.76	9.21
Mammography			
MLO	100% (27/27)^b^	5.69	5.64
CC	96.0% (24/25)^b^	6.59	6.18

a Of the 41 patients with axillary metastatic lymph nodes and marker clips + ^125^I seed placed in breast tumors, 8 patients had breast oblique mammogram feasible for calculation of ΔS of ^125^I seed in lymph nodes.

b Calculation of ΔS of ^125^I seed in breast tumors was feasible in 27 and 25 cases respectively.

MLO, mediolateral oblique; CC, craniocaudal; ^※^S, Average of relative distances of ^125^I seed from the marking clip; ΔS, Difference in relative distance of ^125^I seed from the marker clip before and after NAT.

### The FNR and negative predictive values for different tracers

The overall FNR for the application of dye tracer TAD, radionuclide tracer TAD, and dual tracer TAD were 5.4, 5.2, and 3.4%, respectively (*P*>0.05). The negative predictive values were 93.0, 93.0, and 95.2%, respectively (*P*>0.05; Table 3). There was not statistically significant difference of FNR between different tracer TAD techniques. Although there was a tendency for the FNR to decrease with dual tracer compared to single tracer TAD techniques, TAD for the dye tracer TAD likewise reduced the FNR to a lower level.

**Table 3:** Overall FNR, sensitivity, negative predictive value, accuracy, and detection rate of TAD after NAT.

Diagnostic values	FNR % (*n*)	sensitivity% (*n*)	NPV % (*n*)	accuracy% (*n*)	Detection rate% (*n*)
TAD (dye+clip)	5.4 (3/56)	94.6 (53/56)	93.0 (40/43)	96.8 (93/96)	97.2 (104/107)
TAD (radioisotope +clip)	5.2 (3/58)	94.8 (55/58)	93.0 (40/43)	96.9 (95/98)	98.1 (105/107)
TAD (dual tracer+clip)	3.4 (2/59)	96.6 (57/59)	95.2 (40/42)	98.0 (97/99)	100.0 (107/107)
*P*	0.820	0.820	0.889	0.824	0.377

FNR, false-negative rate; NPV, negative predictive value; TAD, targeted axillary dissection

#### The FNR of different tracers in different cN stage

The Logistic regression analyses of FNR after NAT was showed in Table 4. There was no significant association between the cN stage and FNR after NAT. The FNRs were 2.7, 5.1, and 2.6% among dye, radionuclide TAD, and dual tracer TAD in cN1 patients, respectively (*P*>0.05). The FNRs were 10.5, 5.3, and 5.0% among dye, radionuclide, and dual tracer TAD in cN2 patients, respectively (*P*>0.05). The FNR guided by the dual tracer TAD technique was significantly lower than the FNR guided by the dye tracer-guided TAD.

**Table 4:** Logistic regression analyses of FNR for different TAD after NAT

	Number of SLN detected				Initial axillary lymph node staging		
Group	1	2	≥3	*P*	cN1	cN2	*P*
TAD (dye)	0(0/11)	9.1% (2/22)	4.4% (1/23)	0.598	2.7% (1/37)	10.5% (2/19)	0.263
TAD (isotope)	0(0/12)	8.7% (2/23)	4.4% (1/23)	0.794	5.1% (2/39)	5.3% (1/19)	0.610
TAD (dual tracer)	0(0/12)	5.6% (1/18)	3.4% (1/29)	0.712	2.6% (1/39)	5.0% (1/20)	0.567

#### Analysis of the number of lymph node detections guided by the TAD technique

There was also no statistically significant difference between the lymph node number detected and the FNR of the applied TAD. The FNR for dye tracer TAD was 0, 9.1%, and 4.4% for 1, 2, and ≥3 lymph nodes detected, respectively (*P*=0.598). The FNR for radionuclide tracer TAD was 0, 8.7%, and 4.4% when detected 1, 2, and ≥3 nodes, respectively (*P*=0.794). The FNR for the dual tracer TAD was 0, 5.6%, and 3.4% when detected 1, 2, and ≥3 nodes, respectively (*P*=0.712). Therefore, TAD technique does not require to detect ≥3 lymph nodes to reduce the FNR.

## Discussion

The FNR of SLNB after NAT can meet clinical needs with dual tracers and detected ≥3 SLNs. However, 56.3% of patients in ACOSOG Z1071 trial^10^ and 34% of patients in SENTINA trial^11^ were unable to achieve the successful detection of three or more SLNs. This could potentially be attributed to tumor cells obstructing the lymphatics and impacting the flow of blue dye or radionuclide. Therefore, it becomes critical to optimize SLNB after NAT to achieve appropriate FNR. In this study, there were no significant differences in FNR, sensitivity, and accuracy among different TAD. The dual tracer TAD technique could decrease the overall FNR to 3.4%. At the same time, there was no statistically significant of FNR after NAT between the different tracer TAD techniques. It is worth noting that the dye tracer TAD technique also demonstrated high accuracy. In patients with 1, 2, and ≥3 lymph nodes detected, the overall FNR with the dye tracer TAD technique was 0, 9.1, and 4.4%, respectively. Therefore, as radioisotope tracer is not approved for clinical application in some country and regions, the dye tracer TAD might be more feasible to assess the status of ALNs after NAT.

TAD technique demonstrated feasibility in assessing the response status of ALN after NAT in breast cancer^12–14^. Currently, there is a lack of consensus on the optimal method and materials for marking lymph nodes. Due to the risk of nondetection about metal marker clip, it is necessary to use guidewire and Carbon tattooing to locate the clipped nodes, and guidewire localization is prone to transverse breakage and displacement. In addition, nanocarbon-stained SLN and blue-stained SLN are similar in appearance and easy to be confused. In a prospective study by Straver *et al*.^15^, ^125^I seeds were used as a novel surgical technique to localize ALN. The seeds were applied in a median operative time of 10 min (range: 6–13 min) with a dose of 0.04–0.19 mCi. This was the first instance of using ^125^I seeds to identify metastatic lymph nodes, successfully targeting and detecting them. In this study, there was no statistically significant in FNR between the different ways of localizing metastatic lymph nodes. We found the detection rate was 82.6% for the application of clip and 100% for the application of ^125^I seeds and (or) combined with clip to localize metastatic ALN. It is worth noting that several companies sell versions of this ^125^I seeds, with the seed typically priced from around $17 to $20. But the cost of a traditional metal marker clip with associated deployment device is ~$50, and the cost of a sonographically visible waterabsorbable clip is ~$60. As can be seen, ^125^I seeds have the advantage of not producing displacement, high detection rates and are inexpensive^16^. Therefore, it is recommended that the placement of ^125^I seeds to localize metastatic lymph nodes in a neoadjuvant setting.

The radiation dose for ^125^I seed application was 0.1–0.3 mCi for this study. While this dose may not have achieved a therapeutic effect, it could potentially impact the assessment of NAT efficacy by targeting tumor cells in the lymph node. If the radiation dose is reduced to a certain level, there is a risk that ^125^I seeds cannot be detected accurately. In addition, the radioactive safety of ^125^I seeds requires authorization from governmental health agencies for certification and shipment of the ^125^I seed source to an authorized radioactive waste company for disposal^17^. Future prospective studies will apply a more rigorous experimental design to evaluate how ^125^I seed TAD technology can benefit the survival of patients exempted from ALND. And we will also investigate the extent to which ^125^I seeds kill tumor cells in the ALN.

This study has certain limitations. Firstly, the most important is the small sample size. Secondly, lacking multicenter external data to verify the accuracy is another limitation in our study. Finally, further exploration is required to confirm long-term survival benefits in breast cancer patients who do not undergo ALND with TAD.

In conclusion, considering inexpensive and detect rate of ^125^I seeds, it is recommended that placement of ^125^I seeds to localize metastatic nodes in neoadjuvant setting. The TAD guided by dye tracer is also feasible for axillary de-escalation surgery after NAT in countries or regions without radiolabeled colloid.

## Ethical approval

Ethical approval for this study (Ethical Committee No. SDTHEC20200324) was provided by the Ethical Committee of Shandong Cancer Hospital Ethics Committee, Shandong, Jinan.

## Consent

Informed consents were obtained from all patients, and all procedures were in accordance with the ethical standards of the responsible institutional committee on human experimentation and with the Helsinki Declaration.

## Source of funding

Shanghai Anti-cancer and Anti-cancer Development Foundation (CYBER-2022-002), China Postdoctoral Science Foundation (Grant No. 2022M721987), National Natural Science Foundation of China (82172873); Natural Science Foundation of Shandong Province (ZR2021QH002); Taishan Scholars Program of Shandong Province (tsqn202211337); The Key Research and Development Program of Shandong (Major Science & Technology Innovation Project) (2021SFGC0501).

## Conflicts of interest disclosure

The authors declare that there is no conflict of interest regarding the publication of this paper. All authors have stated that they have no conflicts of interest in this work.

## Research registration unique identifying number (UIN)

1. Name of the registry: internal mammary sentinel lymph node biopsy in early breast cancer patients with clinically axillary node-negative (IMSLNBCANN).
2. Unique identifying number or registration ID: NCT01668914.
3. Hyperlink to your specific registration (must be publicly accessible and will be checked): https://www.clinicaltrials.gov/.

## Guarantor

The guarantors are Zhao Bi and Yong-Sheng Wang.

## Data availability statement

The datasets generated during and/or analyzed during the current study are available from the corresponding author on reasonable request.
